# Expression profile of immune response genes in patients with Severe Acute Respiratory Syndrome

**DOI:** 10.1186/1471-2172-6-2

**Published:** 2005-01-18

**Authors:** Renji Reghunathan, Manikandan Jayapal, Li-Yang Hsu, Hiok-Hee Chng, Dessmon Tai, Bernard P Leung, Alirio J Melendez

**Affiliations:** 1 Department of Physiology, National University of Singapore, Singapore; 2 Department of Infectious Disease, Tan Tock Seng Hospital, Singapore; 3 Department of Rheumatology, Allergy and Immunology, Tan Tock Seng Hospital, Singapore; 4 Department of General Medicine, Tan Tock Seng Hospital, Singapore

## Abstract

**Background:**

Severe acute respiratory syndrome (SARS) emerged in later February 2003, as a new epidemic form of life-threatening infection caused by a novel coronavirus. However, the immune-pathogenesis of SARS is poorly understood. To understand the host response to this pathogen, we investigated the gene expression profiles of peripheral blood mononuclear cells (PBMCs) derived from SARS patients, and compared with healthy controls.

**Results:**

The number of differentially expressed genes was found to be 186 under stringent filtering criteria of microarray data analysis. Several genes were highly up-regulated in patients with SARS, such as, the genes coding for Lactoferrin, S100A9 and Lipocalin 2. The real-time PCR method verified the results of the gene array analysis and showed that those genes that were up-regulated as determined by microarray analysis were also found to be comparatively up-regulated by real-time PCR analysis.

**Conclusions:**

This differential gene expression profiling of PBMCs from patients with SARS strongly suggests that the response of SARS affected patients seems to be mainly an innate inflammatory response, rather than a specific immune response against a viral infection, as we observed a complete lack of cytokine genes usually triggered during a viral infection. Our study shows for the first time how the immune system responds to the SARS infection, and opens new possibilities for designing new diagnostics and treatments for this new life-threatening disease.

## Background

Severe acute respiratory syndrome (SARS) emerged in 2003, as a new epidemic form of life-threatening infection [1]. As of September 2003, there were 8098 cases of SARS from 29 countries with 774 deaths (WHO). SARS is characterized by high fever, malaise, rigors, headache, dry cough, and progression to interstitial infiltration in lungs with eventual mortality of greater than 10% in many countries [2]. SARS has been shown to be caused by a novel coronavirus; SARS-CoV, with genome sequences recently published [3-7]. However, the pathogenesis of SARS is poorly understood. Major hematological features of this disease are lymphopenia, transient thrombocytopenia, and normal neutrophil and monocyte counts [8]. It has been shown that SARS coronavirus infects and replicates in a wide variety of host cells, including PBMCs, in susceptible animals and human beings [9,10]. Hence, to understand the host response to this pathogen, we profiled the gene expression patterns of peripheral blood mononuclear cells (PBMC) from SARS patients, compared to healthy controls using oligo nucleotide microarrays. We found that in the PBMC from SARS patients a number of genes were differentially expressed, as compared to healthy controls, including immune-related genes and these genes are not the typical ones expected in a viral infection.

During a viral infection, most cell types in the body respond by secreting high levels of type 1 interferons (IFN-α and IFN-β) [11]. IFN-α/β can directly induce antiviral activities in neighboring cells, preventing viral spread by increasing the resistance of uninfected cells toward the virus. Moreover, these IFNs can activate Natural Killer (NK) cells mediated cytotoxity toward virus-infected cells [11,12], and there is accumulating evidence that IFN-α/β contribute to driving the adaptive-immune response in the T helper cell type 1 (Th1) direction, via stimulation of IFN-γ expression [12]. NK cells can produce IFN-γ [13], which activates leukocytes, such as monocytes/macrophages, that, in turn, participate in the antiviral responses by producing free radicals and proinflammatory cytokines such as TNF-α [13]. During the response to viral infections, a key role is played by the expansion and activation of CD4+ and CD8+ T cells, which are central to the antiviral immunity, including their capability to inhibit replication and clear the infection. CD8+ cells have a direct effector role through cytotoxic T lymphocyte mediated lysis, and cytokine and chemokine production [14]. The role of CD4+ T cells in antiviral immunity is highly dependent on production of cytokines, notably IFN-γ [15], and the cytolytic activity exerted by a subset of CD4+ T cells [16]. Activation, coordination, and regulation of the above-described antiviral responses are mediated by complex mechanisms, where cytokines play important roles. However, to our surprise, we found that the patients' response of SARS appears to be mainly an innate inflammatory response, rather than a specific immune response against a viral infection. There is no significant level of up-regulation of MHC-I genes, neither for major cytokines including IFNs, nor for genes involved in complement mediated cytolysis, suggesting that the immune response against SARS-CoV may be different from other viral infections.

## Results and discussion

To study the differential immune-gene expression patterns induced by SARS coronavirus, PBMCs from patients with SARS and normal subjects were examined using microarray technology. We shooed to use PBMCs as these cells are more easily obtained from patients compared to other infected tissues, and the SARS-CoV has recently been shown to infect PBMCs [9,10]. The method of global gene expression analysis using oligonucleotide microarrays has proven to be a sensitive method to develop and refine the molecular determinants of several human disorders, including cancer and autoimmune diseases, and has provided us with signatures of the immune response [17]. Using this technology, complemented with powerful analytical methods, we compared the gene expression profiles of PBMC from a series of SARS patients with those of healthy donors. To ensure the reliability and reproducibility of the microarray analysis, Pearson Correlation factors using the signal from all the normal samples were calculated. All four control arrays have Pearson correlation coefficients (r) of >0.95, which suggests an excellent reproducibility among individual arrays in the same experiment and between normal control experiments.

**Figure 1:**
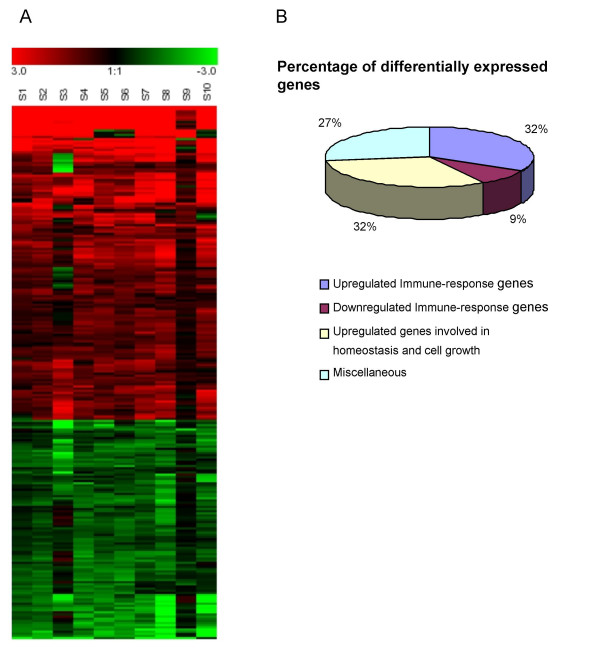
**Clustering and distribution of differentially expressed genes**. **A**. Hierarchical clustering of gene expression data from PBMCs from 10 SARS patients showing different classes of gene expression profiles. Each row represents a separate gene and each column a separate SARS patient. 248 genes have been selected for this analysis which is described in methods. The expression index for each gene (rows) in each sample (column) is indicated by a color code. The color scale ranges from saturated green for log ratios -3.0 and above to saturated red for log ratios 3.0 and above. Red indicates increased gene expression levels, whereas green indicates decreased levels compared with normal samples. **B**. Pie chart showing the percentage distribution of the differentially expressed genes from the PBMCs of 10 SARS patients.

We analyzed the expression pattern of over ~8,700 genes from the PBMCs of 10 SARS patients, and compared the expression patterns with healthy control samples. Student's unpaired T-test (*P *< 0.01) was performed and significant genes were selected. Cluster analysis was performed to find distinct groups of genes that were significantly changed. The criteria used for cluster analysis is different from filter criteria used for differentially expressed genes. 248 genes were selected for Hierarchical clustering (Figure 1) which includes all the 186 differentially expressed genes. The severity of the disease does not seem to change the genetic profile of the PBMCs: 5 of our patients were in intensive care, whereas the other 5 patients did not require intensive care, at the time the blood was taken. However, no substantial gene-expression differences were observed that could correlate to disease severity.

To our surprise, the expression of genes coding for cytokines was completely absent, and the immune-related genes which were overexpressed are usually associated with innate-immune response against bacterial infection and not against a viral infection (Table 1). Several genes were highly up-regulated in patients with SARS, such as, genes coding for Lactoferrin, S100A9 and Lipocalin 2 among others (Table 1). Lactoferrin is a 78- to 80-kDa glycoprotein synthesized by glandular epithelial cells and mature neutrophils, frequently used as a marker for neutrophil degranulation at sites of inflammation, having well recognized bacteristatic and bactericidal properties [18]. It is now clear that the biological actions of lactoferrin are not restricted to its bacteristatic and bactericidal properties. Indeed, a wide array of actions has been reported for lactoferrin, including enhancing NK cell activity, as well as stimulating neutrophil aggregation and adhesion [18]. The myeloid-related proteins, S100A8, S100A9, and S100A12 have been shown to regulate lymphocyte, monocyte, and neutrophil migration and are found in the extracellular milieu during infections and inflammatory episodes [19,20]. The high levels of serum S100A8/A9 in chronic inflammatory pathologies such as rheumatoid arthritis and inflammatory bowel disease, as well as during bronchitis and tuberculosis [20], suggest that they might play a role in inflammatory reactions.

Lipocalins are a family of small, secreted proteins, which have little amino acid sequence homology (20–30%) but share a common three-dimensional structure [21,22]. Tissue distribution studies have revealed that a member of this family 24p3 lipocalin (24p3) is mainly expressed in the liver during an acute phase response [21]. It has also been detected in spleen, lung and the uterus. In the latter location, its expression has been found to be coincident with parturition, a time of major tissue remodeling and inflammation [22]. 24p3 has also been detected in the conditioned media of LPS stimulated murine PU5.1.8 macrophages and therefore it has been suggested to function in defense against infectious agents [22]. However, recent evidence proposed that the lipocalins may trigger apoptosis in immune cells via an unknown cell surface receptor [23]. SARS patients with respiratory distress fulfill criteria for Acute Respiratory Distress Syndrome (ARDS) and diffuse alveolar damage is seen in the lungs on histological examination of postmortem [24]. We speculate that upregulation of lipocalins are part of the host response in limiting unwanted tissue damage and in reducing inflammation and lung fibrosis. In addition, enhanced expression of lipocalins may play a role in the potential cause of lymphopenia observed in the majority of patients.

There is also up-regulation of expression of genes, such us, Bactericidal Permeability Increasing Protein (BPI) and Carcino Embryonic Antigen related Cell adhesion Molecule 8 (CEACAM 8 or CD66b). BPI is released from activated neutrophils and is an endogenous antibiotic, which rapidly kills Gram negative bacteria by high affinity binding to the LPS component of the cell wall [25]. CEACAM 8 is expressed by activated monocytes and granulocytes, and significant up-regulation of this gene indicates the involvement of innate immune cells in SARS. Other genes which encode proteins like Leukotrien-B4 receptor (LTB4R), Leukotrien-A4 hydrolase, IL-8 receptor (IL-8RA), anaphylatoxin C3a receptor-1 (C3aR1), Neutrophil Cytosolic Factor 1 (NCF 1), S100 calcium binding protein A9, Defensin, (DEFA 1/4), LPS binding protein CAP18 (CAMP), and Peptidoglycan Recognition Protein (PGLYRP) are involved in chemotaxis, inflammatory reaction and superoxide metabolism [26]. Similarly, Formyl Peptidase Receptor (FPR) genes are expressed by activated neutrophils. Regulation of expression of FPR on neutrophils plays a key role in neutrophil polarization and chemotaxis. Chemotaxis is effected by a neutrophil membrane mesh, *via *remodeling of the actin component of the membrane [27]. Up-regulation of CD24 and FcγR3A indicates some degree of neutrophil, B cell and NK cell activity. FcγR3a, a low affinity receptor for IgG, expressed in activated macrophages and NK cells, facilitates Antibody Dependent Cell mediated Cytotoxicity (ADCC) by NK cells [13].

There is moderate up-regulation of the kappa light chain of the Nuclear Factor (NFκB 1A) and the B-cell lymphoma 3-encoded protein (BCL3) (Table 1). The major form of NFκB is a heterodimer of p65/p50 subunits, which regulates expression of many proteins in activated immune cells by binding to DNA. It interacts with NFκB through its ankyrin repeats and it favors p50 dimerization by recruiting p50 monomers from the cytoplasmic pool of p105/p50 dimers, and thereby enhancing nuclear translocation and DNA binding of p50 dimers [28]. BLC3 is expressed by activated B cells and T cells on mitotic stimuli, playing an important role in transcription activation [29].

**Figure 2:**
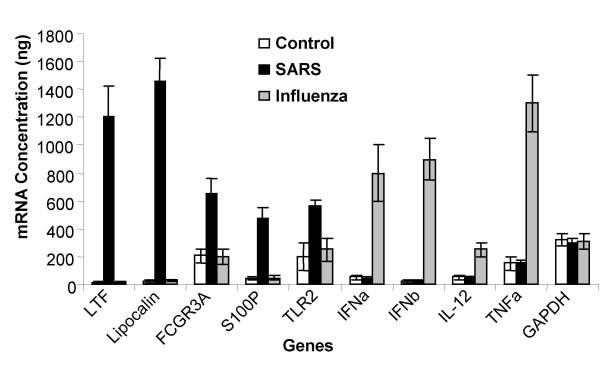
**Real-Time PCR of selected genes**. PBMCs were isolated from 4 healthy volunteers (Control), from 10 SARS infected patients (SARS), and from 5 influenza virus infected patients (Influenza). Total RNA was extracted from all the samples and Light-Cycler Real-Time PCR was performed. The concentrations of these genes mRNA were calculated using respective standard curves. Lactoferrin expression (LTF); Lipocalin expression (Lipocalin); S100P expression (S100P); FCGR3A expression (FCGR3A); TLR2 expression (TLR2); Interferon Alpha expression (IFNa); Interferon Beta expression (IFNb); Interleukin 12-p40 expression (IL-12); Tumor Necrosis Factor Alpha expression (TNFa); and GAPDH expression (GAPDH). Results are expressed as average ± SD of gene expression for each group (control n = 4; SARS n = 10; and influenza n = 5).

In SARS patients there is down-regulation of genes (Table 2) which regulate proliferation and differentiation of T-cells, such as the Lymphocyte-specific protein tyrosine kinase (LCK), which is required for phosphorylation of CD3. There is down-regulation of genes coding for the epsilon polypeptide of the TCR (CD3E), IL-2 induced T-cell kinase (ITK), the Zeta chain of the TCR (CD3Z), the Alpha 4 subunit of VLA-4 receptor (ITGA4), the Chemokine (C-C motif) Receptor 7 (CCR7), and the Interleukin 10 receptor alpha (IL10 RA). All these point to a potential state of unresponsiveness towards the SARS-CoV antigens. It has been described that T cell function in patients with peritonitis had decreased Th1 function, without increased Th2 response, and furthermore T cell proliferation and cytokine secretion correlated with mortality [30]. In our study, there is up-regulation of genes involved in homeostasis and cell growth (Table 3); for example, genes involved in DNA synthesis, nucleosome assembly and protein synthesis, such as, genes encoding Translation Initiation Factor 1A (EIF1 AY), and histone proteins. There is down-regulation of genes coding for negative regulators of the cell cycle, such as, Retinoblastoma-like 2 protein (RBL2), Cyclin-Dependent Kinase Inhibitor 1B (CDKN1B) and anti-apoptoic protein TNF induced protein GG2-1 etc (data not shown). These findings indicate a high degree of proliferation of immune cells; however, there is no significant level of increase in the expression of cytokines like IL-2 or IL-3 and their receptors, which are required for the proliferation and the differentiation of T cells and effector functions of B cells and NK cells. So the increased cell proliferation may be of a granulocyte lineage rather than a monocytic lineage. We confirmed our microarrays findings by Real time PCR on selected upregulated genes such as Lactoferrin Lipocalin, S100P, FCGR3A, and TLR2 (Figure 2). The average expression of all the five genes by real-time RT-PCR was compared to the average expression levels found by microarray analysis (Table 4). The real-time PCR method verified the results of the gene array analysis and showed that those genes that were up-regulated as determined by microarray analysis were found to be up-regulated by real-time PCR analysis. Moreover, since we did not find any upregulation of cytokine-genes usually associated with a viral infection, we selected a few cytokines and compared the SARS samples, with samples collected from 5 influenza virus-infected patients. The observed results, in figure 2, showed that in the influenza patients the mRNA for type I interferons (IFNα and IFNβ), TNFα and IL-12-p40 were upregulated, whereas genes upregulated in the SARS patients (LTF, Lipocalin, S100P, FGR3A and TLR2), were not upregulated in the influenza patients. Therefore, firstly, this confirms that cytokines usually triggered during a viral infection were not detected in the SARS samples (but were triggered in the influenza patients); and secondly, genes triggered in the SARS patients were not triggered in influenza patients.

In this study we have performed extensive analysis of gene expression of PBMCs of SARS patients using a microarray platform that includes more than 8000 gene sequences. However, one potential drawback in our current experimental design is that the gene expression levels were compared between patient samples and normal controls using PBMCs, rather than purified individual cell types. While it would be of interest to determine the exact proportions of monocytes, lymphocytes and contaminating granulocytes in our PBMC preparations, unfortunately, given the highly infective nature of patient samples and lack of suitable flowcytometry facility with appropriate bio-safety control, FACS analysis was, however, not possible.

Our results suggest that the response of SARS affected patients seems to be mainly an innate inflammatory response, rather than a specific immune response against a viral infection. There is no significant level of up-regulation of MHC-I genes or major cytokines including IFNs (α,β, and γ), or genes involved in complement mediated cytolysis, suggesting that the immune response against the SARS-CoV may be different from other viral infections, or that the virus may be using a unusual strategy to evade the host immune system and cause the pathogenesis and mortality.

## Conclusions

This differential gene expression profiling of PBMCs from patients with SARS, strongly suggests that the response of SARS affected patients seems to be mainly an innate inflammatory response, rather than a specific immune response against a viral infection. There is no significant level of up-regulation of MHC-I genes or major cytokines, including IFNs, or complement mediated cytolysis, suggesting that the immune response against the SARS-CoV may be different from other viral infections or that the virus may be using a different strategy to evade the host immune system and cause the pathogenesis and mortality. The severity of the disease does not seem to change the genetic profile of the PBMCs: 5 of our patients were in intensive care, whereas the other 5 patients did not require intensive care, at the time the blood was taken. However, no substantial gene-expression differences were observed that could correlate to disease severity. Moreover, we confirmed (by real-time-PCR) that cytokines usually associated with viral infections (such as interferons and other cytokines) were not detected in the SARS samples, but were triggered in another viral infection (influenza patients). We also showed that genes triggered in the SARS patients were not triggered in the influenza patients.

Our study shows, for the first time, how the immune system responds to the SARS infection and opens new possibilities for designing new diagnostics and treatments for this new life-threatening disease.

## Methods

### Patients and peripheral blood RNA extraction

Our study included ten adult patients (S1-S10) who were diagnosed with SARS according to the World Health Organization (WHO) SARS criteria and admitted to the Tan Tock Seng Hospital, Singapore, for treatment and four additional normal human subjects (C1-C4). Our protocol was approved by the Tan Tock Seng Hospital Research Ethics committee. Peripheral blood was collected by vein-puncture of the brachial vein; mononuclear cells (PBMC) were prepared using histopaque (Sigma- Aldrich, St. Louis, MO) according to manufacturer's recommendations. Sample preparation and processing procedures were carried out as described in the Affymetrix Gene Chip Expression Analysis Manual (Affymetrix Inc., Santa Clara, CA). Briefly, total RNA was extracted from PBMCs, using Trizole method (Invitrogen, Carlsbad, CA) and further purified using RNeasy columns according to manufacturer's instructions (Qiagen, Valencia, CA). Integrity of total RNA was confirmed by formamide gel electrophoresis and quantification was carried out by measuring the A_260 nm_.

### Generation of cDNA and labeled cRNA

5 μg of total RNA was used to synthesize double stranded cDNA using T7-(dT24) oligonucleotide primer and Superscript reverse transcriptase (Invitrogen). The resultant cDNA was purified by phenol:chloroform extraction and ethanol precipitation in presence of 7.5 M ammonium acetate. 1 μg of purified cDNA was subsequently used to synthesize biotin labeled cRNA by *in vitro *transcription (IVT) using T7 RNA polymerase at 37°C for 5-6 hr as per manufacturers' instructions (ENZO labeling kit, Ambion, USA). Labeled cRNA obtained after IVT was purified using RNeasy columns (Qiagen). Purified cRNA was fragmented using fragmentation buffer (40 mmol/L Tris acetate, pH 8.1, 100 mmol/L Potassium acetate, 30 mmol/L Magnesium acetate) at 94°C for 35 min.

### Microarray hybridization and scanning

Fragmented cRNA (10 to11μg/ probe array) was used to hybridize to human focus array (HG-Focus Array) at 45°C for 16 hr with constant rotation of 60 rpm in a Gene chip hybridization oven 640 (Affymetrix). The chips were washed and stained using Gene chip fluidics Station 400 (Affymetrix). Staining was performed using streptavidine phycoerythrin conjugate (SAPE, Molecular Probes, Eugene, OR), followed by the addition of biotinylated antibody to streptavidine (Vector Laboratories, CA), and finally with streptavidine phycoerythrin conjugate. Probe arrays were scanned using Agilent Gene Array Scanner Series US 74900593 (Agilent technologies, USA).

### Data filtering and analysis

An absolute expression analysis was performed using Microarray Suite Software 5.0 (Affymetrix) and relative mRNA expression levels were expressed as plus or minus fold changes compared to normal controls. Each chip was scaled to an overall intensity of 500 to correct for minor differences in overall chip hybridization intensity, and to allow comparison between chips. The data from ~8700 genes was imported in to MicroDB 3.0 and Data Mining Tool 3.0 (Affymetrix) for further analysis. Pearson Correlation coefficient (r) was used to ensure the reproducibility of the data using signal from normal samples. T-test was performed to identify genes that were differentially expressed in SARS patients over normal samples. The statistical significance of the differential expression of any gene was assessed by computing *P *value for each gene. Any gene for which this *P *value was < 0.01 was considered to be differentially expressed. We selected 186 genes for further analysis that met the following criteria:

(i) Changes in expression of at least 2 fold higher or lower comparing the normal

(ii) Signal >500

(iii) Detection *P *< 0.01 and

(iv) Genes which met the above criteria in at least 30% of samples.

Finally, data representing 10 SARS patients fold changes over control samples were averaged.

Genes were annotated according to biological process using the Gene ontology: tool for the unification of biology from the Gene Ontology Consortium [31]. The complete set of raw data was deposited into the NCBIs' Gene Expression Omnibus (GEO) and it can be accessed through the GEO accession 'GSE1739' .

### Hierarchical clustering

Unweighted average linkage Hierarchical clustering was applied for samples using the 'Genesis' software [32]. Genes with 'Present' call (P < 0.01) and significantly changes in expression of at least 2 fold higher or lower comparing the normal were selected. Finally 248 genes which are passing this filter criteria in at least 15% of samples and above were selected for clustering.

### Real-time quantitative PCR

Real-time PCR was performed for ten genes, namely: Lactoferrin (Primers: 5' tcg tcc tgc tgt tcc tcg ggg 3' and 5' tcc agc ggt cct gcg aag gcc 3'); Lipocalin (Primers: 5' aag ccc ctg ctc ctg gcc atc agc 3' and 5' cga cct gat gct gta tgc cac gtg 3'); S100P (Primers: 5' cat gat cat aga cgt ctt ttc 3' and 5' aca cga tga act cac tga agt 3'); TLR2 (Primers: 5' gta tct gca agg gca gct cag gat 3' and 5' ttc ctc aag gaa ggt aag tcc agc 3'); FCGR3A (Primers: 5' ctc cgg ata tct ttg gtg act 3' and 5' tgc aga gca gtg ttc ttc cag 3'); IFNA (Primers: 5'atg gcc ttg acc ttt gct tt 3' and 5'tgg aag att tcc tca tag c 3'); IFNB (Primers: 5'atg acc aac aag tgt ctc ctc caa a 3' and 5' ttc ttc cag gac tgt ctt ca 3'); IL-12 p40 (Primers: 5'atg tgt cac cag cag ttg gtc atc 3' and 5'ctg aat gtc aaa tca gta ct 3'); TNFA (Primers: 5'gag tga caa gcc tgt agc cca tgt tgt agc 3' and 5'gca atg atc cc a aag tag acc tgc cca gac 3'); GAPDH (Primers: 5'acc aca gtc cat gcc atc ac 3' and 5'tcc acc acc ctg ttg ctg ta 3'). For amplicon detection, the Light Cycler RNA Master SYBR Green Kit (Roche) was used as described by the manufacturer. PCRs were performed in a LightCycler^® ^instrument (Roche) as follows: reverse transcription at 61°C for 20 min, initial denaturation at 95°C for 2 min; amplification for 45–65 cycles of denaturation (95°C, 5s, ramp rate 2°C /s), annealing (optimal temperature, 5s, ramp rate 2°C /s) and extension (72°C, product length [bp]/25 s, ramp rate 2°C /s). A single online fluorescence reading for each sample was taken at the end of extension step. Quantitative results were expressed by identification of the second derivative maximum points, which marked the cycles where the second derivatives of the fluorescence signal curves are at maximum. These points were expressed as fractional cycle numbers. Then, these cycle numbers were plotted against the logarithm of the concentrations of serially 2-fold diluted standard samples to obtain a standard curve. The concentrations of unknown samples were calculated by extrapolation from this standard curve. Positive sample specificity was confirmed by determining the melting curve (95°C, 5s, ramp rate 20°C /s; 68°C, 15s, ramp rate 20°C /s; 95 °C, 0s, ramp rate 0.1°C /s, continuous measurement).

## Authors' contributions

RR carried out the sample preparation, hybridization and drafted the manuscript. MJ carried out statistical analysis, data filtration, and data deposition and helped with manuscript preparation. LYH, HHC and DT provided the blood samples and the clinical comments for the study. BPL advised on study design and development. AJM devised and coordinated the study, revised and finalized the manuscript. All authors read and approved the final manuscript.

## Figures and Tables

**Table 1:** Immune-response related genes which were found to be significantly up-regulated in PBMCs of SARS patients. Level of expression is expressed in Fold change (average of fold changes of ten patients, S1-S10) as compared to that of control samples from normal human subjects (C1-C4).

**Gen Bank ID**	**Description**	**Gene name**	**Fold change (S1-S10)**
NM_002343.1	Lactotransferrin	LTF	149.63
M33326.1	Carcinoembryonic antigen-related cell adhesion molecule 8	CEACAM8	97.29
NM_005564.1	Lipocalin 2	LCN2	80.40
NM_004660.2	S100 calcium binding protein A9	S100A9	65.39
NM_001725.1	Bactericidal permeability-increasing protein	BPI	49.94
NM_005091.1	Peptidoglycan recognition protein	PGLYRP	47.86
NM_001925.1	Defensin alpha 4	DEFA4	46.88
U19970.1	Antimicrobial LPS-binding protein CAP18	CAMP	43.36
NM_005980.1	S100 calcium-binding protein P	S100P	39.52
NM_005143.1	Haptoglobin	HP	34.78
NM_004084.2	Defensin alpha 1	DEFA1	29.51
NM_006865.1	Leukocyte immunoglobulin-like receptor, subfamily A, member 3	LILRA3	19.43
NM_002870.1	RAS oncogene family (RAB13)	RAB13	14.85
NM_003064.1	Secretory leukocyte protease inhibitor	SLPI	11.35
NM_000265.1	Neutrophil cytosolic factor 1	NCF1	11.30
NM_000634.1	Interleukin 8 receptor alpha	IL8RA	10.85
AL138717	Rag D protein	RAGD	7.84
BG327863	CD24 antigen	CD24	7.69
NM_000419.2	integrin, alpha 2b (antigen CD41B)	ITGA2B	5.50
NM_001828.3	Charot-Leyden crystal protein	CLC	5.47
AI078167	Nuclear factor of kappa light polypeptide gene enhancer in B-cells inhibitor, alpha	NFKBIA	5.21
NM_005621.1	S100 calcium-binding protein A12	S100A12	5.05
NM_003255.2	Tissue inhibitor of metalloproteinase 2	TIMP2	4.93
NM_002029.1	Formyl peptide receptor 1	FPR1	4.59
NM_005384.1	Nuclear factor, interleukin 3 regulated	NFIL3	4.50
M25915.1	Complement cytolysis inhibitor (CLI)	CLU	4.07
NM_002965.2	S100 calcium-binding protein A9	S100A9	4.05
NM_014214.1	Inositol (myo)-1(or 4)-monophosphatase 2	IMPA2	3.95
NM_003189.1	T-cell acute lymphocytic leukemia 1	TAL1	3.94
U62027.1	C3a receptor	C3AR1	3.83
NM_001150	Alanyl (membrane) aminopeptidase	ANPEP	3.83
BE789881	RAS oncogene family	RAB31	3.75
NM_001769.1	CD9 antigen (p24)	CD9	3.73
NM_014358.1	C-type lectin, superfamily member 9	CLECSF9	3.53
NM_006834.1	RAS oncogene family member (RAB32)	RAB32	3.40
J04162.1	Leukocyte IgG receptor (Fc-gamma-R)	FCGR3A	3.32
NM_006866.1	Lleukocyte immunoglobulin-like receptor, subfamily A2	LILRA2	3.30
U41070.1	Leukotriene b4 receptor	LTB4R	3.23
NM_000632.2	Integrin, alpha M	ITGAM	3.20
BC003393.1	Phosphoinositide-3-kinase, catalytic, beta polypeptide	PIK3CB	3.13
NM_002432.1	Myeloid cell nuclear differentiation antigen	MNDA	3.08
NM_003264.1	Toll-like receptor 2	TLR2	3.05
NM_004475.1	Flotillin 2	FLOT2	3.04
NM_003461.1	Zyxin	ZYX	2.96
NM_003897.1	Immediate early response 3	IER3	2.91
AF119873.1	Serine (or cysteine) proteinase inhibitor alpha-1	SERPINA1	2.87
NM_016205.1	Platelet derived growth factor C	PDGFC	2.74
NM_005479.1	Frequently rearranged in advanced T-cell lymphomas	FRAT1	2.72
AV756141	Colony stimulating factor 2 receptor beta	CSF2RB	2.71
NM_006019.1	T-cell, immune regulator 1	TCIRG1	2.59
J02959.1	Leukotriene A-4 hydrolase	LTA4H	2.59
BC004188.1	Tubulin, beta, 2	TUBB2	2.57
NM_001780.1	CD63 antigen	CD63	2.53
NM_003254.1	Tissue inhibitor of metalloproteinase 1	TIMP1	2.37
AF070673.1	Stannin	SNN	2.35
BC003570.1	Similar to vesicle-associated membrane protein 3	VAMP3	2.31

**Table 2:** Immune-response related genes which were found to be significantly down-regulated in PBMCs of SARS patients. Level of expression is expressed in Fold change (average of fold changes of ten patients, S1-S10) as compared to that of control samples from normal human subjects (C1-C4).

**Genbank ID**	**Description**	**Gene name**	**Fold change (S1-S10)**
D13720.1	IL2-inducible T-cell kinase	ITK	3.64
AF288571.1	Lymphoid enhancer factor-1	LEF1	3.63
NM_005356.1	Lymphocyte-specific protein tyrosine kinase	LCK	3.20
NM_003151.1	Signal transducer and activator of transcription 4	STAT4	3.14
BG435404	ADP-ribosylation factor-like 7	ARL7	2.99
J04132.1	T cell receptor zeta-chain	CD3Z	2.96
BG532690	Alpha 4 subunit of VLA-4 receptor	ITGA4	2.91
NM_001838.1	Chemokine (C-C motif) receptor 7	CCR7	2.83
AI743792	Sialyltransferase 1	SIAT1	2.71
NM_001558.1	Interleukin 10 receptor, alpha	IL10RA	2.71
BF669455	Sialomucin	CD164	2.58
NM_000733.1	Epsilon polypeptide of CD3	CD3E	2.58
AI073984	Interferon consensus sequence binding protein 1	ICSBP1	2.48
NM_005475.1	Lymphocyte adaptor protein	LNK	2.38
AB014560.	Ras-GTPase activating protein SH3 domain-binding protein 2	G3BP2	2.31
NM_004757.1	Small inducible cytokine subfamily E, member 1	SCYE1	2.19

**Table 3:** Genes involved in homeostasis and cell growth, which were found to be significantly up-regulated in PBMCs of SARS patients. Level of expression is expressed in Fold change (average of fold changes of ten patients, S1-S10) as compared to that of control samples from normal human subjects (C1-C4).

**Gen Bank ID**	**Description**	**Gene name**	**Fold change (S1-S10)**
BC005248.1	Eukaryotic translation initiation factor 1A	EIF1AY	44.79
NM_001008.1	Ribosomal protein S4, Y-linked	RPS4Y	20.75
NM_001062.1	transcobalamin I (vitamin B12 binding protein, R binder family)	TCN1	16.88
NM_002357.1	MAX dimerization protein	MAD	11.88
NM_012387.1	Peptidyl arginine deiminase, type V	PADI4	9.12
NM_000184.1	Hemoglobin, gamma G	HBG2	8.7
NM_000559.1	Hemoglobin, gamma A	HBG1	8.1
NM_004653.1	SMC (mouse) homolog, Y chromosome	SMCY	7.07
NM_002934.1	Ribonuclease, RNase A family, 2	RNASE2	5.99
M60721.1	Homeo box 1	HLX1	5.85
NM_005178.1	B-cell CLLlymphoma 3	BCL3	5.11
NM_001964.1	Early growth response 1	EGR1	4.9
NM_006931.1	Solute carrier family 2, member 3	SLC2A3	4.89
NM_002863.1	Phosphorylase, glycogen	PYGL	4.89
NM_021122.2	Fatty-acid-Coenzyme A ligase, long-chain 2	FACL2	4.87
L13974.1	Nuclear factor (erythroid-derived) 2	NFE2	4.77
AI313324	H2A histone family, member O	HIST2H2AA	4.48
BC002649.1	H1 histone family, member 2	HIST1H1C	4.44
AF073310.1	insulin receptor substrate-2	IRS2	4.43
NM_005330.2	hemoglobin, epsilon 1	HBE1	3.97
AF073890.1	Cathepsin X precursor	CTSZ	3.69
AB002559.1	syntaxin binding protein 2	STXBP2	3.69
NM_017445.1	H2B histone family, member S	HIST1H2BK	3.64
NM_003118.1	Secreted protein, acidic, cysteine-rich (osteonectin)	SPARC	3.62
AL353759	Two genes for novel histone 1	HIST1H2AC	3.54
NM_003681.1	Pyridoxal (pyridoxine, vitamin B6) kinase	PDXK	3.54
BC001886.1	Ribonucleotide reductase M2 polypeptide	RRM2	3.53
AI082078	Actinin, alpha1	ACTN1	3.42
AF087942.1	Glycogenin-1L	GYG	3.39
AL538601	Sjogren syndrome antigen A2	SSA2	3.36
NM_001805.1	CCAATenhancer binding protein	CEBPE	3.26
NM_003528.1	H2B histone family, member Q	HIST2H2BE	3.13
BC001906.1	Similar to metaxin 1	MTX1	3.11
NM_006755.1	Transaldolase 1	TALDO1	3.07
BC000893.1	Histone family, member A	HIST1H2BK	3.06
AL161952.1	Glutamate-ammonia ligase	GLUL	2.99
BC002842.1	H2B histone family, member B	HIST1H2BD	2.78
NM_004219.2	Pituitary tumor-transforming 1	PTTG1	2.75
NM_012228.1	Pilin-like transcription factor	PILB	2.73
L12711.1	Transketolase	TKT	2.68
BE561479	Glyceraldehyde-3-phosphate dehydrogenase	GAPD	2.61
NM_012331.2	Methionine sulfoxide reductase A	MSRA	2.6
M33197	Glyceraldehyde-3-phosphate dehydrogenase	GAPD	2.59
NM_005195.1	CCAAT enhancer binding protein	CEBPD	2.57
NM_018840.1	Putative Rab5-interacting protein	C20orf24	2.45
AA314406	Thyroid Hormone Receptor Associated protein	TRAP95	2.44
NM_004832.1	Glutathione-S-transferase like; glutathione transferase omega	GSTTLp28	2.42
NM_013272.2	Solute carrier family 21 (organic anion transporter)	SLC21A11	2.42

**Table 4:** Expression of microarray analysis compared with Real-time RT-PCR analysis

**Gene name**	**Microarray**		**Realtime RT-PCR**	
	
	Mean expression*	*Fold increase*	mRNA conc.(ng)*	*Fold increase*
LTF	12403.95/83.78	148.05	1204/13	92.6
Lipocalin	9788/135.94	72	1455/25	58.2
FCGR3A	6202.81/1885.35	3.29	650/205	3.17
S100P	7496.57/292.49	25.63	475/45	10.5
TLR2	3459.33/1141.69	3.03	563/200	2.8

*Calculated as the average of 10 SARS samples over (/) the average of the 4 normal controls, and expressed as fold increase.
